# Minimally Invasive Surfactant Therapy Using a 2.0 mm Uncuffed Endotracheal Tube as the Conduit: An Easily Adaptable Technique

**DOI:** 10.7759/cureus.5428

**Published:** 2019-08-19

**Authors:** Karthikeyan Gengaimuthu

**Affiliations:** 1 Neonatology and Pediatrics, International Modern Hospital, Dubai, ARE

**Keywords:** prematurity, minimally invasive surfactant therapy, respiratory distress syndrome, barotrauma, endotracheal intubation

## Abstract

Minimally invasive surfactant therapy (MIST) is the accepted way of surfactant administration in Europe, and in 2018, we reported its successful outcome with three babies in Dubai. Although this procedure minimizes the barotrauma associated with intubation for surfactants, threading the fine infant-feeding tube is perceived to be technically difficult. Semi-rigid catheters like the angiocath and the less invasive surfactant administration (LISA) catheter simplify the procedure. We have used successfully the smallest size of endotracheal tube available (2.0 mm) as a surrogate LISA catheter in our neonatal unit in Dubai. We present herein the simplicity and ease of this procedure versus the conventional LISA or MIST technique.

## Introduction

Early initiation of continuous positive airway pressure (CPAP), right from the delivery room, prevents alveolar collapse and the resultant atelectotrauma. Nasal CPAP is the mainstay of the currently recommended noninvasive respiratory support strategies for preterm neonates [1]. Worsening of the disease process needs an escalation of support, which includes surfactant administration, conventional mechanical ventilation, advanced modalities of ventilation like high-frequency oscillation, and extracorporeal membrane oxygenation (ECMO), in that order [2]. As the surfactant has to be delivered onto the alveolar sacs and distal airways, babies were intubated and thereafter ventilated for surfactant therapy until the early years from 2000 to 2010 [3]. The need felt for minimizing the time spent on mechanical ventilation to reduce the barotraumas and oxygen toxicity led to the INSURE (INtubate, SURfactant, and Extubate) technique for surfactant administration wherein post-surfactant administration, the babies were extubated to nasal CPAP as soon as feasible [1-4]. Early surfactant administration has proven advantages over delayed administration, and fraction of inspired oxygen (FIO2) requirements exceeding 0.4 are generally a valid point for intervention; many would consider this earlier on, from 0.3 to 0.4, depending on the clinical status of the neonate [1].

Minimally invasive surfactant therapy (MIST) or less invasive surfactant administration (LISA) was evolved to administer surfactants, avoiding intubation while continuing noninvasive respiratory support through the nasal CPAP throughout the procedure [1-2]. The four ways of MIST that have been described in the literature are a) tracheal instillation using a thin flexible feeding tube assisted by Magill forceps or using semirigid catheters, b) pharyngeal instillation of surfactant, c) nebulizing the surfactant, and d) using a laryngeal mask airway. Of these, the tracheal instillation technique is widely adapted and this is what is referred to by the term LISA in Europe. By definition, only the nebulized aerosol administration of surfactants can be termed as truly ‘noninvasive’ [5]. LISA using an infant feeding tube needs suitably designed Magill forceps for guiding into the trachea and is technically more difficult to learn than conventional intubation. LISA using semirigid catheters, of which the LISA catheter is the prototype, is easier to practice and doesn’t need Magill forceps [6-7]; it fared better than an angiocath in a manikin study [8].

Although we had earlier done MIST using a thin feeding catheter eight times in our Dubai neonatal units without much technical difficulty, we found that using the LISA catheter is technically easy and avoids even the brief deterioration in the clinical status of the neonate noted earlier on with the thin feeding catheter technique (desaturation and bradycardia). The availability and procurement of the LISA catheter and angiocath is difficult due to logistic issues. Considering this and the cost factor, we bench-rehearsed the use of the smallest endotracheal (ET) tube available, the 2.0 mm one whose inner diameter is equivalent to the infant feeding tube of size 8 F. The adapter of the ET tube is removed and the hub of the syringe barrel directly attached to the outer end of the ET tube for the instillation of the surfactant. Recently, we performed MIST by this direct instillation technique two times in a 34-weeks premature neonate with hyaline membrane disease. The steps of this procedure are described herein, along with the actual video of the procedure.

## Technical report

This procedure was performed on a 34-week female neonate with a birth weight of 2.81 kg, who, despite being on nasal CPAP from birth for respiratory distress syndrome (RDS), was worsening with an increasing oxygen requirement to administer surfactant (Survanta, Abbot Laboratories, Illinois, US). This baby required surfactant administration initially at around 11 hours of age and a second dose was required at 22 hours of age, and on both these occasions, the surfactant was administrated by the MIST technique described below.

MIST procedure 

**Figure 1:**
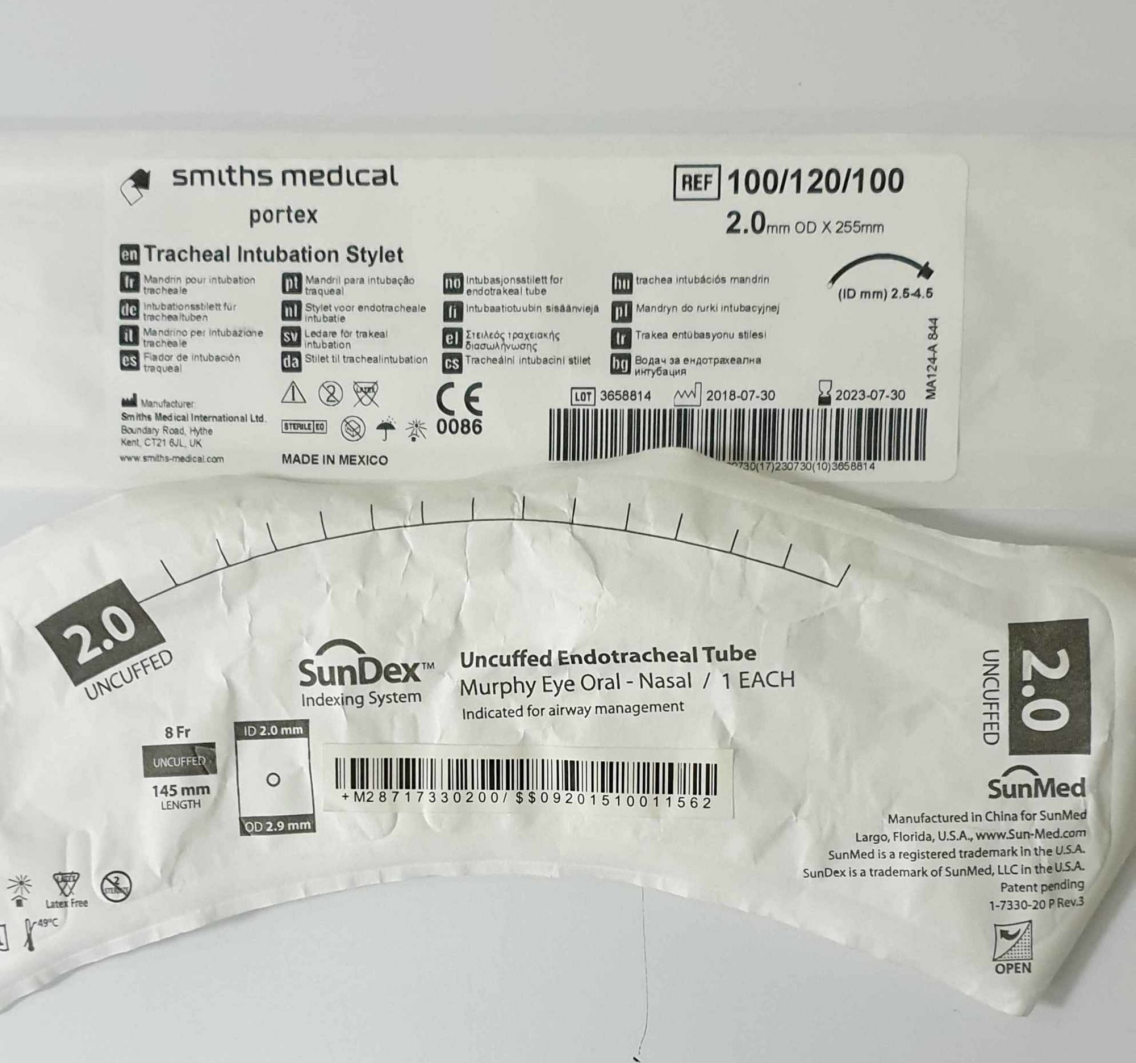
The endotracheal tube and stylet used for the procedure

1. Nasal CPAP was continued throughout the procedure, with adjustment in FIO2, as needed to maintain oxygen saturation.

2. Survanta was loaded in a 10-ml Luer lock syringe after agitating the suspension in the usual manner.

3. A 2.0-mm uncuffed ET tube of inner diameter 2 mm, outer diameter 2.9 mm, 8 F, 145 mm length (Sunmed Largo, Florida, US) and a tracheal intubation stylet, with a 2.0 mm outer diameter x 255 mm (Smiths Medical International Ltd, Kent, UK), were kept ready. The adapter of the ET tube was removed and checked for a tight fit to the hub of the 10 ml Luer lock syringe (Figure 1).

4. After clearing the oral secretions, the glottic area was visualized, the 2.0 mm ET tube inserted (with facilitation by a stylet as needed), and the position confirmed by auscultation.

5. Survanta was administered via the ET tube by attaching the Luer lock end of the 10-ml syringe with Survanta followed by an air flush in aliquots of 10 ml to disperse and deliver Survanta onto the deeper airways.

6. Post the Survanta administration, appropriate FIO2 adjustments were done.

The baby made a rapid recovery following the second dose of Survanta at 22 hours of life, coming off nasal CPAP and then remaining without oxygen from the second day post-procedure.

## Discussion

The whole concept of MIST is based on the surmise that tracheal instrumentation is harmful, and going by the biological plausibility clause of proof of causation, this harm has to be (or can be) quantitative as well qualitative [9]. Quantitative means the duration of instrumentation and qualitative means the size and material of the instrument used for the instillation of surfactant. Endotracheal intubation increases the chances of ventilator-associated pneumonia (VAP) by the following known mechanisms [10-11]:

1. Aspiration of oropharyngeal secretions during the process of intubation.

2. Sledging of the sludge down across the ET tube lumen.

3. Impairment of mucociliary blanket clearance mechanism.

4. But by far the most relevant factor described is the biofilm formation and bacterial colonization inside the lumen. Biofilms are the constant nidi predisposing to bacterial shred versus shed. The shredding and shedding of biofilm layers spread the bacteria down across, resulting in VAP.

5. The endotracheal cuff facilitates the down spread of organisms from the nasopharynx by leakage across the cuff folds.

Major and minor faults: is ET intubation the major fault that needs to be avoided entirely?

The answer currently available in the literature suggests it is not necessarily so, as the chance of ventilator-associated pneumonia and biofilm formation happens usually after the fourth day of intubation [12]. But having an ET tube in situ means frequent suctioning of the ET tube, which further adds to the trauma to the airways and, hence, we should endeavor to shorten the duration of airway violation by the endotracheal tube as much as we can [12]. But it is a fault line that can perpetuate itself to a clinical conundrum by virtue of loss of cough reflex, dependency on the tube, need for prolonged mechanical ventilation, VAP, etc. [13-14]. Hence, the MIST technique of surfactant delivery confers definite clinical benefits to tiny neonates with RDS, facilitating a seamless transition to room air.

The endotracheal tubes and gastric feeding tubes or other indwelling catheters used in neonatal units are, in general, made up of any one of these three materials: polyvinyl chloride (PVC), polyurethane (PU), and silicon [15]. PVC is the thickest and gives a rigid consistency that facilitates the ease of insertion. Hence, traditionally, ET tubes are made of PVC with the incorporation of a radio-opaque line. PU (that is 40 times thinner than PVC for similar strength) cuffed ET tubes are logistically better for the pediatric and adult populations, as trans-cuff micro-aspiration of oropharyngeal secretions is minimized [14,16].

The 5 F infant feeding tube that is used for traditional less invasive surfactant administration is made of PU material and hence lacks the firmness needed for easy maneuvering into the trachea under direct laryngoscopy. Intubation of the trachea is made easier by the relative firmness of the ET tube and its modifications for LIST: the angiocath and LISA catheter that are of 5 F size [8]. The polymer content of the latter ones used for LISA differs from PVC as well as the flexible PU used in infant feeding tubes [8,16]. A 2.0-mm ET tube is 8 F equivalent and hence a 1.5-mm ET tube is 6 F equivalent and by extending this logic, a 5 F equivalent will be a 1.25 mm ET tube that is not available in the market. Can the beneficial neonatal outcomes reported in neonates receiving surfactant by MIST (improvement in the combined outcome of death and/or chronic lung disease) versus INSURE be achieved with ET tubes of relevant sizes, say a 1.5 mm or 1.25 mm ET tube, or with a 2.0 ET tube coupled with the threading a 5 F or 4 F infant feeding tube as during conventional surfactant administration is a research question. Based upon the available evidence, the answer could possibly be yes, as factors other than just the ET intubation play a key role in relevant beneficial outcomes, the most important being the duration of intubation [12].

## Conclusions

MIST can be safely and effectively performed using the smallest diameter ET tube currently available commercially, that is, of a 2.0-mm inner diameter, 8 F equivalent. This is comparatively easier and quicker than conventional MIST using an infant feeding tube otherwise known as LISA.

This can effectively substitute for LISA with an angiocath catheter that has been reported to be technically superior to performing LISA conventionally with a 5 F infant feeding tube. The caveat here is that as the smallest ET tube currently available, the 2.0-mm ET tube is equivalent to the 8 F infant feeding tube and hence it is suitable only for preterm babies of 2 kg and above.

## References

[1] Sweet DG, Carnielli V, Greisen G (2019). European Consensus Guidelines on the management of respiratory distress syndrome - 2019 update. Neonatology.

[2] Gengaimuthu K (2018). Should minimally invasive surfactant therapy be a must in neonatal intensive care units? Pilot report of initial cases in Dubai. Cureus.

[3] Bohlin K, Henckel E, Blennow M (2008). International perspectives. NeoReviews.

[4] Blennow M, Bohlin K (2015). Surfactant and noninvasive ventilation. Neonatology.

[5] Barkkuff WD, Soll RF (2019). Novel surfactant administration techniques: will they change outcome?. Neonatology.

[6] Lista G, Bresesti I, Fabbri L (2018). Is less invasive surfactant administration necessary or ‘only’ helpful or just a fashion. Am J Perinatol.

[7] Rigo V, Debauche C, Maton P, Broux I, Van Laere D (2017). Rigid catheters reduced duration of less invasive surfactant therapy procedures in manikins. Acta Paediatrica.

[8] Fabbri L, Klebermass-Schrehof K, Aguar M (2018). Five-country manikin study found that neonatologists preferred using the LISAcath rather than the angiocath for less invasive surfactant administration. Acta Pædiatrica.

[9] Rothman KJ, Greenland S (2005). Causation and causal inference in epidemiology. Am J Public Health.

[10] Deem S, Treggiari MM (2010). New endotracheal tubes designed to prevent ventilator-associated pneumonia: do they make a difference?. Respir Care.

[11] Gibbs K, Holzman IR (2012). Endotracheal Tube: Friend or Foe? Bacteria, the Endotracheal Tube, and the Impact of Colonization and Infection. Semin Perinatol.

[12] Friedland DR, Rothschild MA, Delgado M, Isenberg H, Holzman I (2001). Endotracheal tube: friend or foe? Bacteria, the endotracheal tube, and the impact of colonization and infection. Arch Otolaryngol Head Neck Surg.

[13] Deem S, Treggiari MM (2010). New endotracheal tubes designed to prevent ventilator associated pneumonia. Do they make a difference?. Respir Care.

[14] Rouze A, Jaillette E, Poissy J, Preau S, Nseir S (2017). Tracheal tube design and ventilator associated pneumonia. Respir Care.

[15] Wallace T, Steward D (2014). Gastric tube use and care in the NICU. Newborn and Infant Nursing Reviews.

[16] Pittiuruti M (2018). Silicon vs polyurethane in 2018. Poster presented at the World Congress Vascular Access, Copenhagen, Denmark.

